# Supplementary material for: State space methods for phase amplitude coupling analysis
Source: Sci Rep. 2022 Sep 24;12:15940. doi: 10.1038/s41598-022-18475-3 (PMC9509338; doi:10.1038/s41598-022-18475-3)
Supplement: Supplementary file 1 — Supplementary Information. [file 41598_2022_18475_MOESM1_ESM.pdf]

Supplementary: State Space Methods for Phase Amplitude Coupling Analysis

Supplementary Section .1 Derivations

Power Spectral Density for the State Space Oscillator Process

In this section, we derive the parametric expression for the power spectral density (PSD) of an oscillation $x^j_{1,t}$ by building an autoregressive moving average process (ARMA) with the same spectral content. For convenience, we will drop the index j in what follows. First, we note that an oscillation is asymptotically second order stationary. Let us compute its autocovariance sequence. Since $\mathscr{R}$ is a rotation matrix $\mathscr{R}(\omega)^k = \mathscr{R}(k\omega)$ and $\mathscr{R}\mathscr{R}^\intercal = I$. Therefore, from equation (1), it comes:

$$\begin{aligned}\mathbb{E}(x_t {x_t}^\intercal) =& a\mathscr{R}(\omega)\mathbb{E}(x_{t-1}{x_{t-1}}^\intercal)(a\mathscr{R}(\omega))^\intercal + Q \\ =& Q\sum_{t=0}^{N} a^{2t} \\ =& Q/(1-a^2) + \mathscr{O}(a^{2N})\end{aligned} \tag{34}$$

and:

$$\begin{aligned}\mathbb{E}(x_{t+k}{x_t}^\intercal) =& a\mathscr{R}(\omega)\mathbb{E}(x_{t+k-1}x_t) \\ =& a^k\mathscr{R}(k\omega)Q/(1-a^2) + \mathscr{O}(a^{2N+k})\end{aligned} \tag{35}$$

We can hence write, for $t = 1..N$, $k = 0..N-t$, $s_{t,t+k} = \mathbb{E}(x_{1,t+k}{x_{1,t}}^\intercal) = s_k$. As a consequence, an oscillation can be approximated by a second order stationary process, and in virtue of the Wiener-Khinchin theorem[38], its theoretical power spectral density is:

$$\mathscr{S}(f) = \lim_{N\to\infty}\frac{1}{F_s}\left(s_0 + 2\sum_{t=1}^{N} s_t e^{-2i\pi t f/F_s}\right) \tag{36}$$

We now consider the ARMA(2,1):

$$\tilde{x}_t = \phi_1\tilde{x}_{t-1} + \phi_2\tilde{x}_{t-2} + \tilde{u}_t + \psi_1\tilde{u}_{t-1},\ \tilde{u}_t \sim \mathscr{N}(0,\tilde{\sigma}^2) \tag{37}$$

to which we impose, for $t = 1..N$, $k = 0..N-t$: $\mathbb{E}(\tilde{x}_t\tilde{x}_{t+k}^\intercal) = s_k$. It follows that:

$$\begin{aligned}s_k =& \phi_1 s_{k-1} + \phi_2 s_{k-2}, k > 2 \\ s_1 =& \frac{\phi_1 s_0 + \psi_1\tilde{\sigma}^2}{1-\phi_2} \\ s_0 =& \phi_1 s_1 + \phi_2 s_2 + \tilde{\sigma}^2(1 + \phi_1\psi_1 + \psi_1^2)\end{aligned} \tag{38}$$

Taking:

$$\begin{aligned}\phi_1 =& 2a\cos(\omega) \\ \phi_2 =& -a^2\end{aligned} \tag{39}$$

satisfies the first equality of equation (38). The remaining conditions can then be rewritten:

$$\begin{aligned}\psi_1\tilde{\sigma}^2 &= -a\sigma^2\cos(\omega) \\ 0 &= \psi_1^2 + \frac{1+a^2}{a\cos(\omega)}\psi_1 + 1\end{aligned} \tag{40}$$

from which we deduce 2 two negative roots $\psi_1^{\pm}$ ultimately leading to the same autocovariance series. Overall, we choose:

$$\begin{aligned}\psi_1 &= -\frac{1}{2}\left(\frac{1+a^2}{a\cos(\omega)} + \sqrt{\left(\frac{1+a^2}{a\cos(\omega)}\right)^2 - 4}\right)\\ \tilde{\sigma}^2 &= -\frac{a\sigma^2\cos(\omega)}{\psi_1}\end{aligned} \tag{41}$$

Applying the discrete Fourier transform to equation (37) yields:

$$\mathscr{F}[\tilde{x}](f)(1-\phi_1 e^{-2i\pi f/Fs} - \phi_2 e^{-4i\pi f/Fs}) = \mathscr{F}[\tilde{u}](f)(1+\psi_1 e^{-2i\pi f/Fs}) \tag{42}$$

since $\tilde{u}_t$ is Gaussian noise, $\mathbb{E}(\tilde{u}_t \tilde{u_{t+k}}^\intercal) = \delta_k \tilde{\sigma}^2$. Therefore, our ARMA(2,1) PSD is:

$$\mathscr{S}[\tilde{x}](f) = \frac{\tilde{\sigma}^2}{F_s}\frac{|1+\psi_1 e^{-2i\pi f/Fs}|^2}{|1-\phi_1 e^{-2i\pi f/Fs} - \phi_2 e^{-4i\pi f/Fs}|^2} \tag{43}$$

Finally, the PSD of an oscillation centered in f_0 is ;

$$\mathscr{S}[x_{1,t}](f) = \frac{\tilde{\sigma}^2}{F_s}\frac{|1+\psi_1 e^{-2i\pi f/Fs}|^2}{|(ae^{-2i\pi f/Fs} - e^{-2i\pi f_0/Fs})(ae^{-2i\pi f/Fs} - e^{+2i\pi f_0/Fs})|^2} \tag{44}$$

An Expectation-Maximization (EM) Algorithm for Independent and Harmonic Oscillation Decompositions

Since all noise terms are assumed to be additive Gaussian, the complete data log likelihood for one time window of length N is:

$$\begin{aligned}\log L &= \log\, p(x_1,...,x_N,y_1,...,y_N)\\ &\mathrel{\hat{=}} -\frac{N}{2}\log|Q| - \frac{1}{2}\sum_{t=1}^{N}(x_t-\Phi x_{t-1})^\intercal Q^{-1}(x_t-\Phi x_{t-1})\\ &\quad -\frac{N}{2}\log|R| - \frac{1}{2}\sum_{t=1}^{N}(y_t-Mx_t)^\intercal R^{-1}(x_t-Mx_t)\end{aligned} \tag{45}$$

We wish to maximize $\log L$ with respect to $\Theta = (\Phi, Q, R)$ but we do not have access to the hidden oscillations x_t. We use an expectation maximization algorithm to alternatively and iteratively estimate (E-Step) and maximize (M-Step) the log likelihood. At iteration r, we use the Kalman filter to estimate x_t given a set Θ_r which gives us access to:

$$G_r(\Theta) = \mathbb{E}_r(\log L|\{y_t\}_{t=1}^N) \tag{46}$$

Then, we deduce Θ_{r+1}:

$$\Theta_{r+1} = \underset{\Theta}{\operatorname{argmax}}\ G_r(\Theta) \tag{47}$$

Kalman Filter and Fixed Interval Smoother Estimates

We use the Kalman filter to estimate the hidden oscillations given the observations and the model parameters. They first predict the state at the next time point, then compare that prediction to the observation, and finally produce an updated estimate based on the most recently observed data. Given the full observation time series, we can apply backward smoothing to refine the update to account for the full observation series (i.e., fixed interval smoothing).

For $t, t_1, t_2 = 1..N$, we note:

$$x_t^N = \mathbb{E}_r(x_t|\{y_t\}_{t=1}^N),\ P_{t_1,t_2}^N = \text{cov}_r(x_{t_1}, x_{t_2}|\{y_t\}_{t=1}^N),\ \text{and } P_t^N = P_{t,t}^N \tag{48}$$

and compute those quantities using the forward smoothing algorithm:

$$\begin{aligned}
\textbf{Prediction}: \ & x_t^{t-1} = \Phi x_{t-1}^{t-1} \\
& P_t^{t-1} = \Phi P_{t-1}^{t-1} \Phi^\intercal + Q \\
\textbf{Kalman Gain}: \ & K_t = P_t^{t-1} M^\intercal (M P_t^{t-1} M^\intercal + R)^{-1} \\
\textbf{Update}: \ & x_t^t = x_t^{t-1} + K_t (y_t - M x_t^{t-1}) \\
& P_t^t = P_t^{t-1} - K_t M P_t^{t-1}
\end{aligned} \tag{49}$$

and a set of backward recursions[50]. For $t = N..1$, and $t_1 < t_2$:

$$\begin{aligned}
\textbf{Backward Gain}: \ & J_{t-1} = P_{t-1}^{t-1} \Phi^\intercal (P_t^{t-1})^{-1} \\
\textbf{Smoothing}: \ & x_{t-1}^N = x_{t-1}^{t-1} + J_{t-1}(x_t^N - \Phi x_{t-1}^{t-1}) \\
& P_{t-1}^N = P_{t-1}^{t-1} + J_{t-1}(P_t^N - P_t^{t-1}) J_{t-1}^\intercal \\
\textbf{Covariance}: \ & P_{t_1,t_2}^N = J_{t_1} P_{t_1+1,t_2}^N
\end{aligned} \tag{50}$$

E-Step

Here we describe $G_r(\Theta)$ for the state space oscillation decomposition with harmonic components. An single oscillation is defined by a rotation matrix $\mathscr{R}(\omega)$, a scaling parameter a and a process noise covariance matrix $Q = \sigma^2 I_{2\times 2}$. In what follows, we consider d independent oscillations, which are respectively associated to $h_1, ..., h_d$ harmonics. For $j = 1..d$, an oscillation with fundamental frequency ω_j is the sum of $h = 1...h_j$, harmonics respectively defined by $\mathscr{R}(h\omega_j)$, $a_{j,h}$ and $Q_{j,h} = \sigma_{j,h}^2 I_{2\times 2}$. We note $D = \sum_{j=1}^d h_j$ the total number of oscillatory components.

For $V \in \mathbb{R}^{2D\times 2D}$, $j = 1..d$ and $h = 1..h_j$, we note $V_{j,h}$ the 2 by 2 diagonal block associated with the h_j^{th} harmonic of oscillation j. Φ and Q are block diagonal matrices whose diagonal blocks are $a_{j,h}\mathscr{R}(h_{j,h})$ and $Q_{j,h}$:

$$\Phi = \text{diag}\left(a_{1,1}\mathscr{R}(\omega_1), ...a_{1,h_1}\mathscr{R}(h_1\omega_1), ..., a_{d,1}\mathscr{R}(\omega_d), ...a_{d,h_d}\mathscr{R}(h_d\omega_d)\right) \tag{51}$$

$$Q = \text{diag}\left(Q_{1,1}, ...Q_{1,h_1}, ..., Q_{d,1}, ...Q_{d,h_d}\right) \tag{52}$$

Additionally, we will use $M = [1 \quad 0 \quad 1 \quad 0 \quad ... \quad 0] \in \mathbb{R}^{2D}$ and for $U \in \mathbb{R}^{2\times 2}$, we note: $\text{rt}(U) := U_{21} - U_{12}$ and $\text{tr}(U) = U_{11} + U_{22}$.

Taking the conditional expectation of the log likelihood $\log L$ at iteration r for a fixed set of parameter $\Theta_r = (\Phi, Q, R)_r$, we obtain[23]:

$$\begin{aligned}
G(\Phi, Q, R)_r \ \hat{=}& -\frac{N}{2}\log|Q| - \frac{1}{2}\text{tr}\left(Q^{-1}(C - B\Phi^\intercal - \Phi B^\intercal + \Phi A \Phi^\intercal)\right) \\
& -\frac{N}{2}\log|R| - \frac{1}{2}\text{tr}\left(R^{-1}(\sum_{t=1}^N (y_t - Mx_t^N)(y_t - Mx_t^N)^\intercal + MP_t^N M^\intercal\right) \\
\hat{=}& \ G(\Phi, Q)_r + G(R)_r
\end{aligned} \tag{53}$$

where:

$$\begin{aligned}
A &= \sum_{t=1}^N (P_{t-1}^N + x_{t-1}^N (x_{t-1}^N)^\intercal) \\
B &= \sum_{t=1}^N (P_{t,t-1}^N + x_t^N (x_{t-1}^N)^\intercal) \\
C &= \sum_{t=1}^N (P_t^N + x_t^N (x_t^N)^\intercal)
\end{aligned} \tag{54}$$

M-Step

We can maximize G_r with respect to R and (Φ, Q) independently. We have:

$$\frac{\partial G_r}{\partial R}(R_{r+1}) = 0 \iff R_{r+1} = \frac{1}{N}\sum_{t=1}^{N}\left((y_t - Mx_t^N)^2 + MP_t^N M^\intercal\right) \tag{55}$$

Since Q is (block) diagonal, we can write:

$$\operatorname{tr}(Q^{-1}V) = \sum_{j=1}^{d}\sum_{h=1}^{h_j}\operatorname{tr}(Q_{j,h}^{-1}V_{j,h}) = \sum_{j=1}^{d}\sum_{h=1}^{h_j}\frac{1}{\sigma_{j,h}^2}\operatorname{tr}(V_{j,h}) \tag{56}$$

A is symmetric and Φ is a block diagonal matrix whose element are 2×2 rotation matrices, we develop equation (53) and obtain:

$$\begin{aligned} G_r(\Phi, Q) &\mathrel{\mathring{=}} -N\sum_{j=1}^{d}\sum_{h=1}^{h_j}\log \sigma_{j,h}^2 \\ &-\sum_{j=1}^{d}\sum_{h=1}^{h_j}\frac{1}{2\sigma_{j,h}^2}\left[\operatorname{tr}(C_{j,h}) - 2a_{j,h}(\operatorname{tr}(B_{j,h})\cos(h\omega_j) + \operatorname{rt}(B_{j,h})\sin(h\omega_{j,h})) + a_{j,h}^2\operatorname{tr}(A_{j,h})\right] \end{aligned} \tag{57}$$

Differentiating with respect to process noises covariances $\sigma_{j,h}^2$, scaling parameter $a_{j,h}$ and fundamental frequencies ω_j yields:

$$\begin{cases} \frac{\partial G}{\partial a_{j,h}}(\Phi, Q) = 0 & \iff a_{j,h}\operatorname{tr}(A_{j,h}) = \operatorname{tr}(B_{j,h})\cos(h\omega_j) + \operatorname{rt}(B_{j,h})\sin(h\omega_j) \\ \frac{\partial G}{\partial \sigma_{j,h}^2}(\Phi, Q) = 0 & \iff \sigma_{j,h}^2 = \frac{1}{2N}\left(\operatorname{tr}(C_{j,h}) - a_{j,h}^2\operatorname{tr}(A_{j,h})\right) \\ \frac{\partial G}{\partial w_j}(\Phi, Q) = 0 & \iff \sum_{h=1}^{h_j}\frac{h}{\sigma_{j,h}^2}\left[a_{j,h}(tr(B_{j,h})\sin(h\omega_j) - rt(B_{j,h})\cos(h\omega_j))\right] = 0 \end{cases} \tag{58}$$

We inject the upper equations of (58) into the third one and we note:

$$\tilde{\omega}_{j,h} = \frac{1}{h}\tan^{-1}\left(\frac{\operatorname{rt}(B_{j,h})}{\operatorname{tr}(B_{j,h})}\right) \text{ and } S_{jh} = \frac{2\operatorname{tr}(A_{j,h})\operatorname{tr}(C_{j,h})}{\operatorname{rt}(B_{j,h})^2 + \operatorname{tr}(B_{j,h})^2} - 1 \tag{59}$$

Using trigonometric identities, equations (58) can finally be rewritten:

$$\begin{cases} \frac{\partial G}{\partial a_{j,h}}(\Phi, Q) = 0 & \iff a_{j,h}\operatorname{tr}(A_{j,h}) = \operatorname{tr}(B_{j,h})\cos(h\omega_j) + \operatorname{rt}(B_{j,h})\sin(h\omega_j) \\ \frac{\partial G}{\partial \sigma_{j,h}^2}(\Phi, Q) = 0 & \iff \sigma_{j,h}^2 = \frac{1}{2N}\left(\operatorname{tr}(C_{j,h}) - a_{j,h}^2\operatorname{tr}(A_{j,h})\right) \\ \frac{\partial G}{\partial a_{j,h}}(\Phi, Q) = 0 & \iff \sum_{h=1}^{h_j}\frac{h\sin(2h(\omega_j - \tilde{\omega}_{j,h}))}{S_{jh} - \cos(2h(\omega_j - \tilde{\omega}_{j,h}))} = 0 \end{cases} \tag{60}$$

Overall, for $j = 1..d$, if $h_j > 1$, we numerically solve for ω_j using equation (60) and deduce $a_{j,h}$ and $\sigma_{j,h}^2$ for $h = 1..h_j$.

If $h_j = 1$, we immediately have:

$$\begin{aligned} w_j &= \tan^{-1}\left(\operatorname{rt}(B_j)/\operatorname{tr}(B_j)\right) \\ a_j &= \frac{\sqrt{\operatorname{rt}(B_j)^2 + \operatorname{tr}(B_j)^2}}{\operatorname{tr}(A_j)} \\ \sigma_j^2 &= \frac{1}{2N}\left(\operatorname{tr}(C_j) - a_j^2\operatorname{tr}(A_j)\right) \end{aligned} \tag{61}$$

Linear Regression: priors, hyperparameters, and normalizing constants

As in[47], we use $\tau_\beta = \sigma_\beta^{-2}$ and we assume that the likelihood of the model defined in equation (21) is:

$$p(A^{\mathrm{f}}, \phi^{\mathrm{s}} | \beta, \tau_\beta) \propto \tau_\beta^{-N/2} \exp\left(-\frac{\tau_\beta}{2} (A^{\mathrm{f}} - X(\phi^{\mathrm{s}})\beta)^\intercal (A^{\mathrm{f}} - X(\phi_t^{\mathrm{s}})\beta) \right) \mathbb{1}_{\{\beta \in W(\overline{K})\}} \tag{62}$$

where $\{A_t^{\mathrm{f}}\}_t = A^{\mathrm{f}}$ and $\{\phi_t^{\mathrm{s}}\}_t = \phi^{\mathrm{s}}$. The conjugate prior is:

$$p(\beta, \tau_\beta) \propto \tau_\beta^{\frac{\tilde{v}+3}{2}-1} \exp\left(-\frac{\tau_\beta}{2} \left[\tilde{b}\tilde{v} + (\beta - \tilde{\beta})^\intercal \tilde{V} (\beta - \tilde{\beta}) \right] \right) \mathbb{1}_{\{\beta \in W(\overline{K})\}} \tag{63}$$

We choose prior hyperparameters $\tilde{V}, \tilde{b}\ \tilde{v}$ and $\tilde{\beta} = [\tilde{\beta}_0\ \tilde{\beta}_1\ \tilde{\beta}_2]^\intercal$ to convey as little information as possible on the phase and the strength of the modulation. Marginalizing equation (63) over τ_β, yields a truncated multivariate t-distribution:

$$p(\beta) \propto \left(1 + \frac{1}{\tilde{v}} (\beta - \tilde{\beta})^\intercal (\tilde{V}/\tilde{b}) (\beta - \tilde{\beta}) \right)^{-\frac{\tilde{v}+3}{2}} \mathbb{1}_{\{\beta \in W(\overline{K})\}} \tag{64}$$

$\tilde{v} \geq 3$ insures that the multivatiate-t variance is defined. It is: $\tilde{v}(\tilde{v}-2)^{-1}\tilde{b}\tilde{V}^{-1}$. Then, we consider the independent random variables $\tilde{A}$, $\tilde{K}$ and $\tilde{\phi}$ such that:

$$\begin{aligned} &\tilde{A} \sim \Gamma(A_0/c, c) \\ &\tilde{K} \sim \mathrm{Uniform}(0,1) \\ &\tilde{\phi} \sim \mathrm{Uniform}(-\pi, \pi) \end{aligned} \tag{65}$$

We note $\gamma = \begin{bmatrix} \tilde{A} & \tilde{A}\tilde{K}\cos(\tilde{\phi}) & \tilde{A}\tilde{K}\sin(\tilde{\phi}) \end{bmatrix}^\intercal$, use $\tilde{b} = 1$, $\tilde{v} = 3$ and we define $\tilde{\beta}$, and $\tilde{V}$ such that:

$$\tilde{\beta} = \mathbb{E}(\gamma) \text{ and } \tilde{V}^{-1} = \mathrm{Cov}(\gamma)(\tilde{v}-2)/\tilde{v} \tag{66}$$

Additionally, we notice that if $A_t^{\mathrm{f}} \approx A_0[1 + K^{\mathrm{mod}} \cos(\phi_t^{\mathrm{s}} + \phi^{\mathrm{mod}})]$ and since $\mathbb{E}_s(\langle \cos(\phi_t^s + \phi^{\mathrm{mod}}) \rangle_t) = 0$ (where $\mathbb{E}_s$ represents an average over trials or windows and $\langle . \rangle_t$ is a temporal average across a given window), $\mathbb{E}_s(\langle A_t^{\mathrm{f}} \rangle_t) = A_0$ and $\langle A_t^{\mathrm{f}} \rangle_t \in [0, 2A_0]$. Therefore, it is reasonable to use $A_0 = \langle A_t^{\mathrm{f}} \rangle_t$ and $c = 1$. Overall:

$$\begin{aligned} &\tilde{v} = 3\ ,\ \tilde{V} = \langle A_t^{\mathrm{f}} \rangle_t^{-1} \begin{pmatrix} 3 & 0 & 0 \\ 0 & 12 & 0 \\ 0 & 0 & 12 \end{pmatrix} \\ &\tilde{\beta} = \begin{bmatrix} \langle A_t^{\mathrm{f}} \rangle_t \\ 0 \\ 0 \end{bmatrix} \text{ and } \tilde{b} = 1 \end{aligned} \tag{67}$$

Posterior parameters are then given by:

$$\begin{aligned} &v = \tilde{v} + N\ ,\ V = \tilde{V} + X(\phi^{\mathrm{s}})^\intercal X(\phi^{\mathrm{s}}) \\ &\overline{\beta} = V^{-1}\left(\tilde{V}\tilde{\beta} + X(\phi^{\mathrm{s}}) A^{\mathrm{f}} \right)\ ,\ b = (\tilde{v}\tilde{b} + H)/v \end{aligned} \tag{68}$$

Where:

$$\begin{aligned} \hat{\beta}_{OLS} &= (X(\phi^{\mathrm{s}})^\intercal X(\phi^{\mathrm{s}}))^{-1} X(\phi^{\mathrm{s}})^\intercal A^{\mathrm{f}} \text{ and:} \\ H &= (A^{\mathrm{f}} - X(\phi^{\mathrm{s}})\hat{\beta}_{OLS})^\intercal (A^{\mathrm{f}} - X(\phi^{\mathrm{s}})\hat{\beta}_{OLS}) \\ &\quad + (\hat{\beta}_{OLS} - \overline{\beta})^\intercal X(\phi^{\mathrm{s}})^\intercal X(\phi^{\mathrm{s}}) (\hat{\beta}_{OLS} - \overline{\beta}) \\ &\quad + (\tilde{\beta} - \overline{\beta})^\intercal \tilde{V} (\tilde{\beta} - \overline{\beta}) \end{aligned} \tag{69}$$

We deduce the posterior distribution:

$$p\left(\beta,\tau_\beta|\{A^{\mathrm{f}}_t,\phi^{\mathrm{s}}_t\}_t\right)=\frac{1}{\overline{Z}}\tau_\beta^{-\frac{\nu+3}{2}-1}\exp\left(-\frac{\tau_\beta}{2}\left[b\nu+(\beta-\overline{\beta})^\intercal V(\beta-\overline{\beta})\right]\right)\mathbb{1}_{(W\beta>0)} \tag{70}$$

The normalizing constant $\overline{Z}$ is obtained by integration and is:

$$\overline{Z}=\frac{\Gamma(\nu/2)(2\pi)^{3/2}}{|V|^{1/2}(\nu b/2)^{\nu/2}}\mathrm{P}(\beta\in W(\overline{K})) \tag{71}$$

where $\mathrm{P}(\beta\in W(\overline{K}))$ is computed using the multivariate t-distribution of parameters $\nu,\overline{\beta}$ and $b^{-1}V$.

Finally, we deduce equation (24) by marginilizing equation (70) over τ_β and have:

$$Z=\frac{\Gamma(\nu/2)}{\Gamma((\nu+3)/2)}\frac{(\nu\pi)^{3/2}}{|V/b|^{1/2}}\times\mathrm{P}(\beta\in W(\overline{K})) \tag{72}$$

Note that for large samples it might be useful to use:

$$\frac{\Gamma((\nu+3)/2)}{\Gamma(\nu/2)}=\left(\frac{\nu+1}{2}\right)\left(\frac{\nu}{2}\right)^{1/2}\left(1-\frac{1}{4\nu}+\frac{1}{32\nu^2}+\frac{1}{128\nu^3}+\mathscr{O}\left(\frac{1}{\nu^4}\right)\right) \tag{73}$$

Second State-Space

In this section, for a given AR order p, we estimate the parameters R_β, Q_β and $\{h_k\}_{k=1}^p$ defined equation (6). Let $C_m=\mathbb{E}(\beta_T^{\mathrm{SSP}}\beta_{T-m}^{\mathrm{SSP}\intercal})$ be the autocovariance sequence of the modulation vectors estimated with equation (25). We have:

$$C_m=\sum_{k=1}^p h_kC_{m-k}+R_\beta(\delta_{0,m}-h_m)+Q\delta_{0,m} \tag{74}$$

where $\delta_{i,j}$ is the the Kronecker delta. Equation (74) can be rewritten:

$$\begin{cases} C_0 = \sum_{k=1}^p h_kC_k+Q \\ \begin{bmatrix}C_1\\C_2\\\vdots\\C_p\end{bmatrix} = \begin{bmatrix}C_0-R_\beta & C_1 & \dots & C_{p-1}\\ C_1 & C_0-R_\beta & \dots & C_{p-2}\\ \vdots & \vdots & \ddots & \vdots\\ C_{p-1} & C_1 & \dots & C_0-R_\beta\end{bmatrix}\begin{bmatrix}h_1\\h_2\\\vdots\\h_p\end{bmatrix}\end{cases} \tag{75}$$

For an observation noise candidate R^*_β, if we can invert equation (75), we immediately access Q^*_β and $\{h^*_k\}_{k=1}^p$. Using the Kalman Filter, we hence deduce the likelihood of the candidate model as in[22].

Therefore, we note R^m_β the smallest eigenvalue of the Toeplitz matrix $\mathbf{C}=(C_{|i-j|})_{i,j=0..p}$, and, numerically optimize the model likelihood with respect to R^*_β in $(0,R^m_\beta)$, where we know that $(\mathbf{C}-IR^*_\beta)$ is full rank.

From R_β we get Q_β and $\{h_k\}_{k=1}^p$ then, once again, we use the Kalman Filter to estimate β_T^{dSSP}.

Modulogram Equivalence

We derive an approximated parametric modulogram for a window of length $\delta t=N/F_s$ centered in τ. We will use:

$$
\begin{aligned}
l &= f_{\mathrm{s}}\delta t \\
t &= k/F_s \\
\Omega_\tau &= \{t, t \in [\tau - \delta t/2, \tau + \delta t/2]\} \\
\Omega_{\tau,\psi} &= \{t \in \Omega_\tau, \phi_t^s \in [\psi - \frac{\delta\psi}{2}, \psi + \frac{\delta\psi}{2}]\} \\
\tilde{\Omega}_{\tau,\psi} &= \{t \in \Omega_\tau, t\omega_{\mathrm{s}} \in [\psi - \frac{\delta\psi}{2}, \psi + \frac{\delta\psi}{2}]\}
\end{aligned} \tag{76}
$$

For clarity we will use t or k without distinction and we remind that:

$$
A_k^{\mathrm{f}} = A_0[1 + K_\tau^{\mathrm{mod}} \cos(\phi_k^s - \phi_\tau^{\mathrm{mod}})] + \varepsilon_k,\ \varepsilon_k \sim \mathscr{N}(0, \sigma_\beta^2) \tag{77}
$$

$$
\mathrm{PAM}(\tau,\psi) = \frac{\int_{\tau-\delta t/2}^{\tau+\delta t/2} \int_{\psi-\psi\delta/2}^{\psi+\delta\psi/2} A_t^{\mathrm{f}} \delta(\phi_t^{so} - \psi') dt d\psi'}{2\pi \int_{\tau-\delta t/2}^{\tau+\delta t/2} A_t^{\mathrm{f}} dt} = \frac{P_2}{P_1} \tag{78}
$$

Additionally, we assume that:

- for $k \in \Omega_\tau$, $\phi_k^s = \frac{\omega_{\mathrm{s}}}{F_s} k + \eta_k$, where $\mathbb{E}(\eta_k) = 0$, $\mathrm{Var}(\eta_k) = \sigma_\phi^2$ and $\sigma_\phi^2 << \frac{\omega_{\mathrm{s}}}{F_s} < 1$
- and, for simplicity, for $\psi \in [-\pi, \pi]$, for all $h : \mathbb{R}^+ \to \mathbb{R}$ smooth , $\sum_{k \in \Omega_{\tau,\psi}} h(k) \approx \sum_{k \in \tilde{\Omega}_{\tau,\psi}} h(k)$

From the central limit theorem, $\sum_{k \in \Omega_\tau} \varepsilon_k = \mathscr{O}_p(\sqrt{N})$ and $\sum_{k \in \Omega_\tau} \eta_k = \mathscr{O}_p(\sqrt{N})$.

We hence have:

$$
\begin{aligned}
P_1 &= \frac{A_0}{F_s} \sum_{k \in \Omega_\tau} \left(1 + K_\tau^{\mathrm{mod}} \cos\left(\frac{\omega_{\mathrm{s}} k}{F_s} - \phi_\tau^{\mathrm{mod}} + \eta_k\right)\right) + \frac{1}{F_s} \sum_{k \in \Omega_\tau} \varepsilon_k \\
&= \frac{A_0}{F_s} \sum_{k \in \Omega_\tau} \left(1 + K_\tau^{\mathrm{mod}} \cos\left(\frac{\omega_{\mathrm{s}} k}{F_s} - \phi_\tau^{\mathrm{mod}}\right)\right) + \frac{1}{F_s} \sum_{k \in \Omega_\tau} \left(\varepsilon_k - A_0 K_\tau^{\mathrm{mod}} \sin\left(\frac{\omega_{\mathrm{s}} k}{F_s} - \phi_\tau^{\mathrm{mod}}\right) \eta_k + \mathscr{O}(\eta_k)\right) \\
&= \frac{A_0}{F_s} \sum_{k \in \Omega_\tau} \left(1 + K_\tau^{\mathrm{mod}} \cos\left(\frac{\omega_{\mathrm{s}} k}{F_s} - \phi_\tau^{\mathrm{mod}}\right)\right) + \mathscr{O}_p(\sqrt{N})
\end{aligned} \tag{79}
$$

But $\sum_{k \in \Omega_\tau} \cos\left(\frac{\omega_{\mathrm{s}} k}{F_s} - \phi_\tau^{\mathrm{mod}}\right) = \cos\left(\frac{\omega_{\mathrm{s}}}{2F_s}(N-1) - \phi_\tau^{\mathrm{mod}}\right) \frac{\sin(N w_s/(2F_s))}{\sin(\omega_{\mathrm{s}}/(2F_s))} \le \frac{2F_s}{\omega_{\mathrm{s}}}$. Therefore:

$$
P_1 = A_0 \frac{N}{F_s} + \mathscr{O}_p(\sqrt{N}) \tag{80}
$$

On the other hand:

$$
\begin{aligned}
P_2 &= \frac{1}{F_s} \sum_{k \in \Omega_{\tau,\psi}} A_k^{\mathrm{f}} \\
&= \frac{A_0}{F_s} \sum_{k \in \tilde{\Omega}_{\tau,\psi}} \left(1 + K_\tau^{\mathrm{mod}} \cos\left(\frac{\omega_{\mathrm{s}} k}{F_s} - \phi_\tau^{\mathrm{mod}} + \eta_k\right)\right) + \frac{1}{F_s} \sum_{k \in \tilde{\Omega}_{\tau,\psi}} \varepsilon_k
\end{aligned} \tag{81}
$$

But $\sum_{k \in \tilde{\Omega}_{\tau,\psi}} \varepsilon_k = \mathscr{O}_p(\delta\psi\sqrt{l})$ and $l \propto N$ so:

$$
P_2 = \frac{A_0}{F_s} \sum_{k \in \tilde{\Omega}_{\tau,\psi}} \left(1 + K_\tau^{\mathrm{mod}} \cos\left(\frac{\omega_{\mathrm{s}} k}{F_s} - \phi_\tau^{\mathrm{mod}} + \eta_k\right)\right) + \mathscr{O}_p(\sqrt{N}) \tag{82}
$$

Using the same argument as the one detailed above we get:

$$P_2 = \frac{A_0}{F_s} \sum_{k \in \tilde{\Omega}_{\tau,\psi}} \left(1 + K_\tau^{\text{mod}} \cos\left(\frac{\omega_s k}{F_s} - \phi_\tau^{\text{mod}}\right)\right) + \mathscr{O}_p(\sqrt{N}) \tag{83}$$

Additionally:

$$\begin{aligned} &\frac{A_0}{F_s} \sum_{k \in \tilde{\Omega}_{\tau,\psi}} \left(1 + K_\tau^{\text{mod}} \cos\left(\frac{\omega_s k}{F_s} - \phi_\tau^{\text{mod}}\right)\right) \\ &= \frac{A_0}{\delta\psi} \int_{\tau - \frac{\delta t}{2}}^{\tau + \frac{\delta t}{2}} \int_{\psi - \frac{\delta\psi}{2}}^{\psi + \frac{\delta\psi}{2}} \left(1 + K_\tau^{\text{mod}} \cos\left(\omega_s t - \phi_\tau^{\text{mod}}\right)\right) \delta_{\tilde{\Omega}_{\tau,\psi}} dt d\psi + \gamma(w_s/F_s) \end{aligned} \tag{84}$$

Where γ is a function such that $\gamma(x) \xrightarrow[x \to 0]{} 0$. Since:

$$\begin{aligned} &\int_{\tau - \frac{\delta t}{2}}^{\tau + \frac{\delta t}{2}} \int_{\psi - \frac{\delta\psi}{2}}^{\psi + \frac{\delta\psi}{2}} \left(1 + K_\tau^{\text{mod}} \cos\left(w_s t - \phi_\tau^{\text{mod}}\right)\right) \delta_{\tilde{\Omega}_{\tau,\psi}} dt d\psi + \gamma(\omega_s/F_s) \\ =& \frac{L}{\omega_s} \times \int_{\psi - \frac{\delta\psi}{2}}^{\psi + \frac{\delta\psi}{2}} \left(1 + K_\tau^{\text{mod}} \cos\left(\phi' - \phi_\tau^{\text{mod}}\right)\right) d\phi' + \mathscr{O}(1) \\ =& \frac{L\delta\psi}{\omega_s} \left(1 + \frac{\sin(\delta\psi/2)}{\delta\psi/2} K_\tau^{\text{mod}} \cos\left(\psi - \phi_\tau^{\text{mod}}\right)\right) + \mathscr{O}(1) \end{aligned} \tag{85}$$

For $\frac{\omega_s}{F_s}$ "sufficiently small", we can write:

$$P_2 = \frac{A_0 l}{\omega_s} \left(1 + \frac{\sin(\delta\psi/2)}{\delta\psi/2} K_\tau^{\text{mod}} \cos\left(\psi - \phi_\tau^{\text{mod}}\right)\right) + \mathscr{O}_p(\sqrt{N}) \tag{86}$$

Finally:

$$\begin{aligned} \text{PAM}(\tau, \psi) &= \frac{1}{2\pi} \frac{\left(1 + \frac{\sin(\delta\psi/2)}{\delta\psi/2} K_\tau^{\text{mod}} \cos(\psi - \phi_\tau^{\text{mod}}) + \mathscr{O}_p(1/\sqrt{N})\right)}{1 + \mathscr{O}_p(1/\sqrt{N})} \\ &= \frac{1}{2\pi} \left(1 + \frac{\sin(\delta\psi/2)}{\delta\psi/2} K_\tau^{\text{mod}} \cos(\psi - \phi_\tau^{\text{mod}})\right) + \mathscr{O}_p(1/\sqrt{N}) \end{aligned} \tag{87}$$

Supplementary Section .2 Additional Results

In this section we present additional results to support the validation and comparison of our algorithm:

Fig. S.1 is a simulated signal composed of three sinusoidal oscillations (A) where the amplitude of the fastest oscillation (C) was modulated by the phase of the middle one (E). The modulation peaked at an angle of $\pi/3$. Both information criterion (AIC and BIC) select the correct number of component (D) and our model correctly infers the latent components (B-C-E) and the phase amplitude coupling (F).

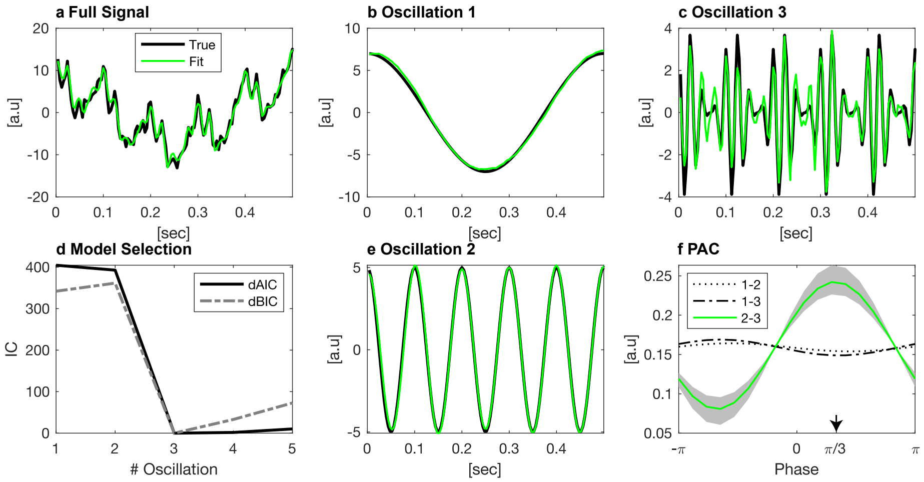

Figure S.1. Model selection and Phase Amplitude Coupling (PAC) in the case of multiple oscillations. The full Signal (A) is decomposed using 3 oscillations (B-C-E) according to model selection criteria (AIC or BIC) (D). Then PAC (F) is estimated by looking at pairs of oscillations. The arrow indicates the true coupling phase between oscillation 2 and 3, which is correctly inferred by our algorithm. Credible intervals are estimated using 100 samples.

Fig. S.2 compares the two methods used in this work in order to estimate spectrograms: (A) multitaper-spectrograms[38] are directly computed from data (Figs. 4, 8) while (B) parametric-spectrograms (Fig. 2) are analytically derived from the model see equation (44).

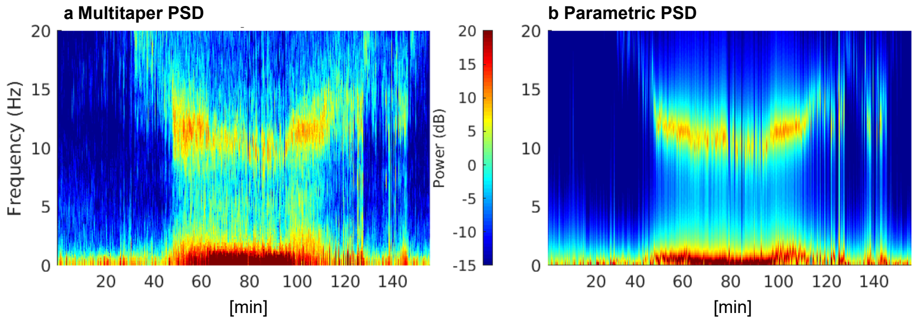

Figure S.2. Comparison between Multitaper (A) and Parametric (B) spectrograms using data from Fig. 2

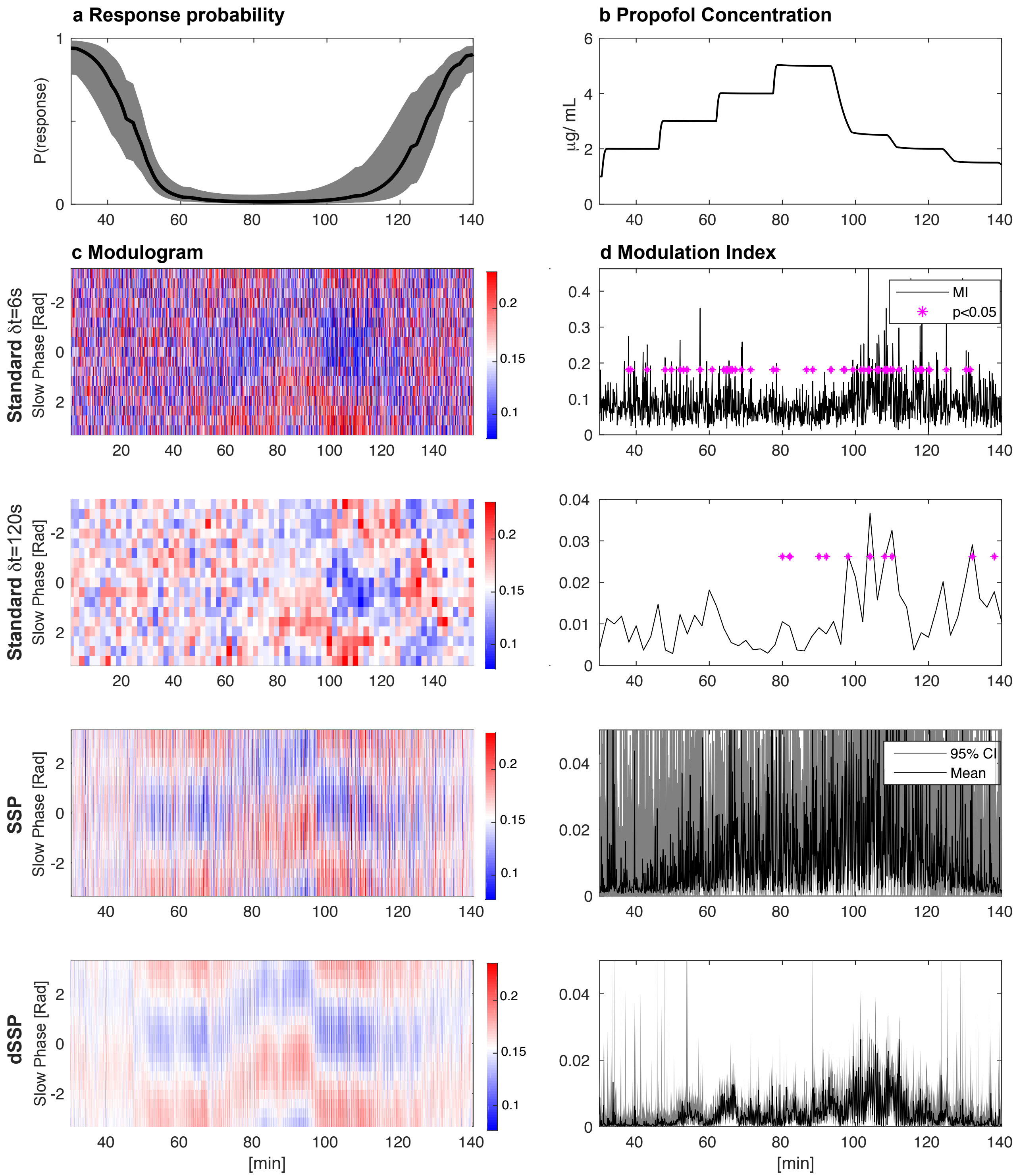

Figure S.3. Phase Amplitude Coupling profile of another subject infused with increasing target effect site concentration of propofol. Left: response probability curves (a) aligned with modulograms (c) (distribution of alpha amplitude with respect to slow phase) computed with standard (top) and parametric (bottom) techniques. Right: propofol infusion target concentration (b) aligned with corresponding Modulation Indices (d). Standard technique significance was assessed using 200 random permutations and CI where estimated using 200×200 samples

Fig. S.3 is the PAC profile of another subject infused with increasing target effect site concentration of propofol.

Fig.S.4 is a comparison of the modulation dynamic estimation between standard analysis and our dSSP.

Fig.S.5 are typical 6s signal traces of signal generated with equation (12).

Fig.S.6, S.7 and S.8 Are the modulation phase (ϕ^{mod}) and frequency (f_s, f_f) recovery estimation and comparison between standard methods, DAR and SSP.

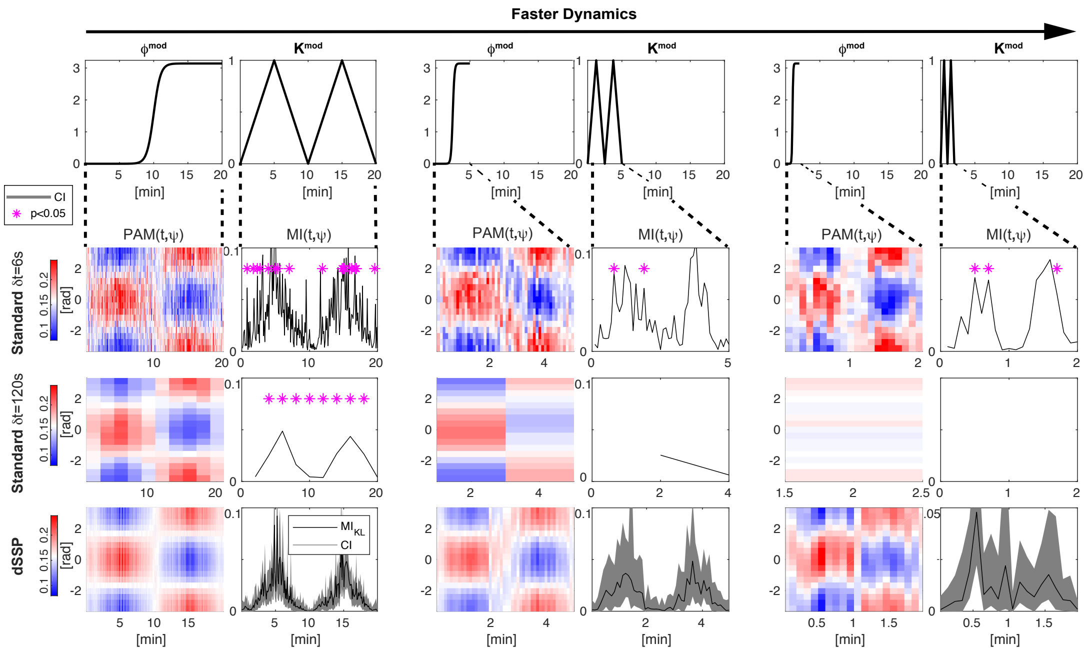

Figure S.4. Comparison of the modulation estimates using standard methods and our new dSSP method. Slow and fast oscillations were generated by filtering white noise around $f_s = 1$Hz and $f_s = 10$Hz with $\Delta f_s^{\text{gen}} = $1Hz and normalized to standard deviation $\sigma_s = 1$ and $\sigma_f = 0.8$. The time scale over which K^{mod} and ϕ^{mod} changed varied between 20 minutes to 5 and 2 minutes. See Fig. S.5 for typical signal traces

.

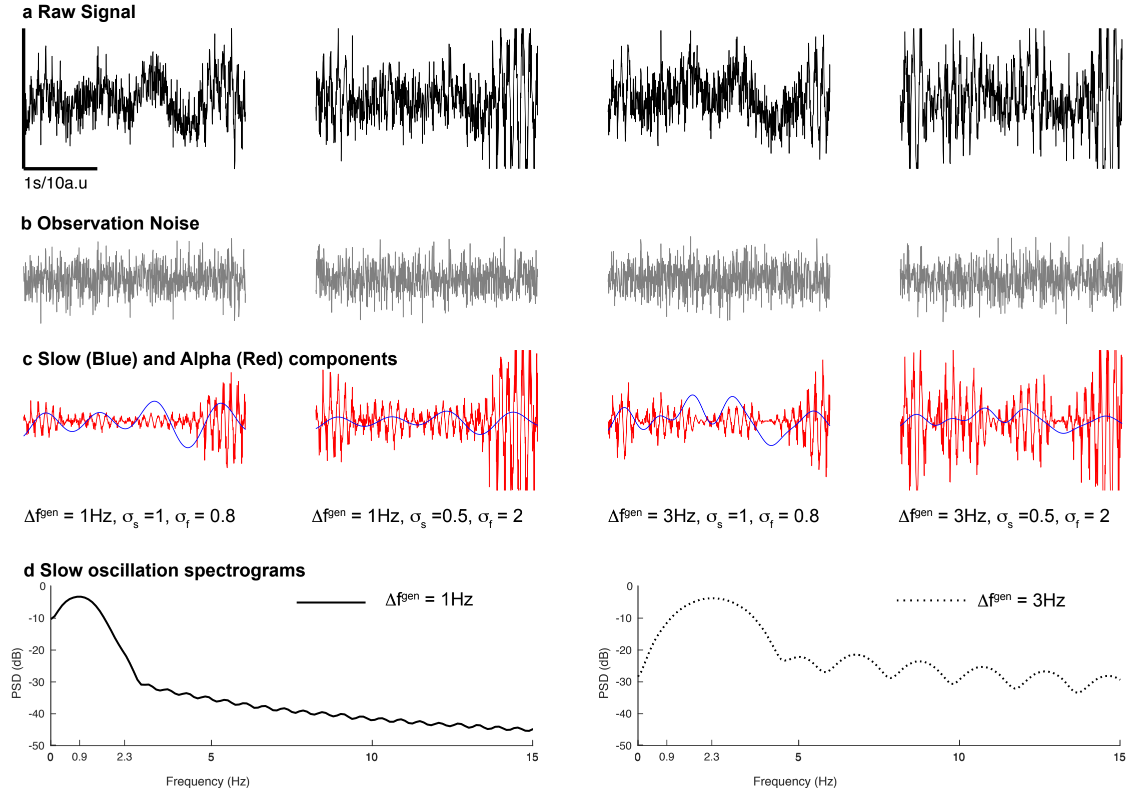

Figure S.5. Typical 6s signal traces generated with equation (12) for 4 different conditions. The raw signal (a) is the sum of the observation noise (b), slow, and alpha oscillations (c). We show slow frequency multitaper PSD (TW=4, K=5 tapers) for different values of $\Delta f_s^{\mathrm{gen}}$ (d).

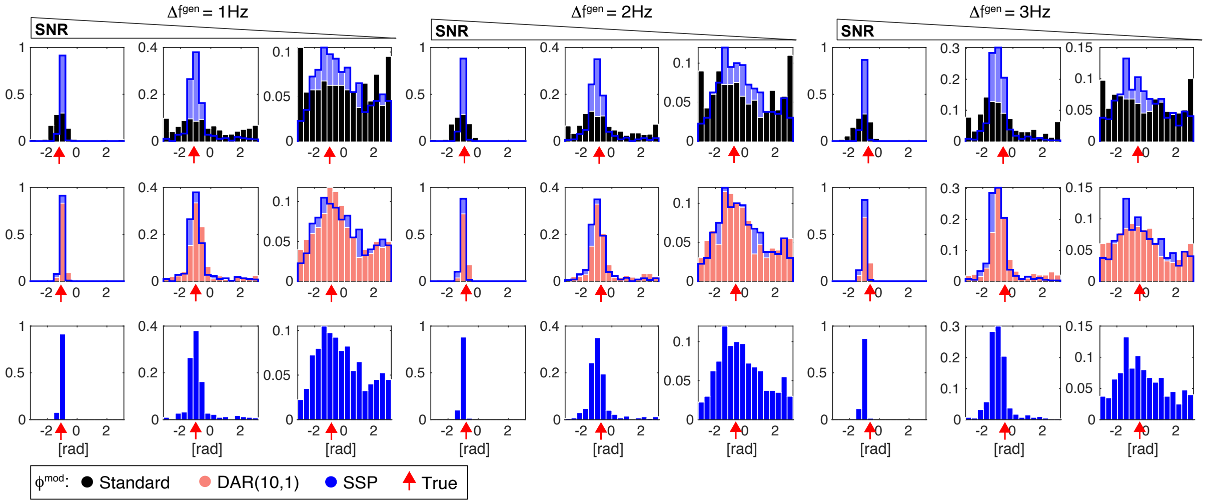

Figure S.6. Modulation phase ϕ^{mod} estimation and comparison between standard methods (black), DAR (pink) and SSP (blue). 400 windows of 2 seconds were generated with: a slow oscillation (filtered from white noise around $f_{\mathrm{s}} = 3$Hz with $\Delta f_{\mathrm{s}}^{\mathrm{gen}}$ and normalized to standard deviation σ_{s}) and a modulated fast oscillation ($\phi^{\mathrm{mod}} = -\pi/3$, modeled with a sinusoid $f_{\mathrm{s}} = 50$Hz and normalized to σ_{f}). We added unit normalized Gaussian noise and we used 3 Signal To Noise Ratio (SNR) conditions ($(\sigma_{\mathrm{s}}, \sigma_{\mathrm{s}}) = (2, 1.5)$, $(1, 0.6)$ and $(0.7, 0.3)$). We show typical signal traces for these different conditions in Fig S.8.

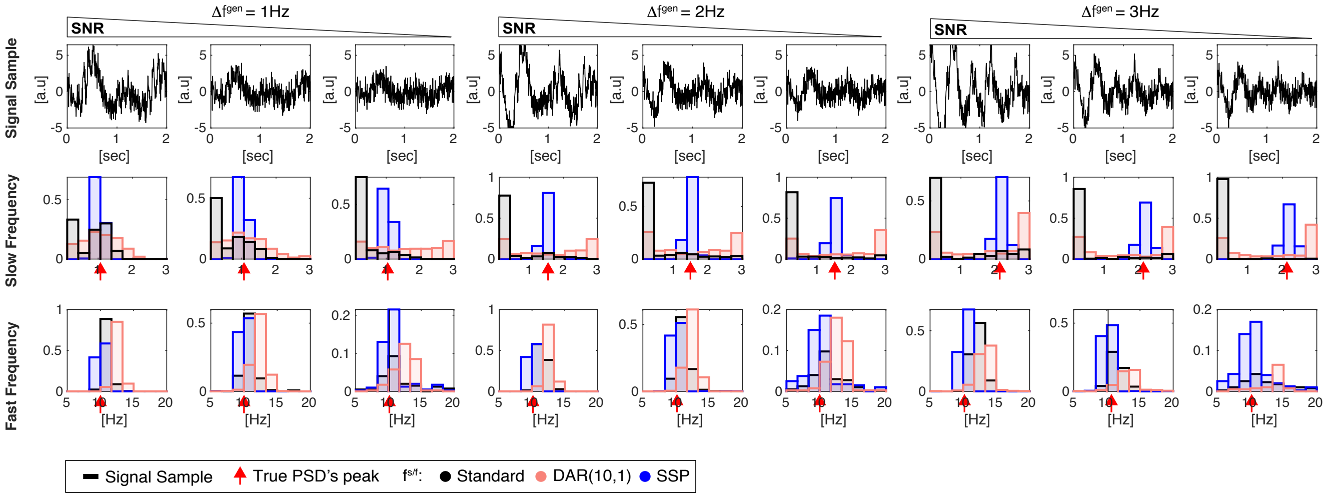

Figure S.7. Slow and Fast oscillation recovery using different algorithms: standard methods (black), DAR (pink) and SSP (blue). 400 windows of 6 seconds were generated with: a slow oscillation (filtered from white noise around $f_s = 1$Hz with bandwidth Δf_s^{gen} and normalized to standard deviation σ_s) and a modulated fast oscillation ($\phi^{\text{mod}} = -\pi/3$, modeled with a sinusoid $f_s = 10$Hz and normalized to σ_f). We added unit normalized Gaussian noise and we used 3 Signal To Noise Ratio (SNR) conditions ($(\sigma_s, \sigma_s) = (2, 1.5)$, $(1, 0.6)$ and $(0.7, 0.3)$). We report typical signal traces for the different conditions (top), slow oscillation recovery alongside the true slow frequency PSD (middle), and fast frequency recovery (bottom). The red arrow indicates the true multitaper PSD (TW=4, K=5 tapers) peak for each oscillation.

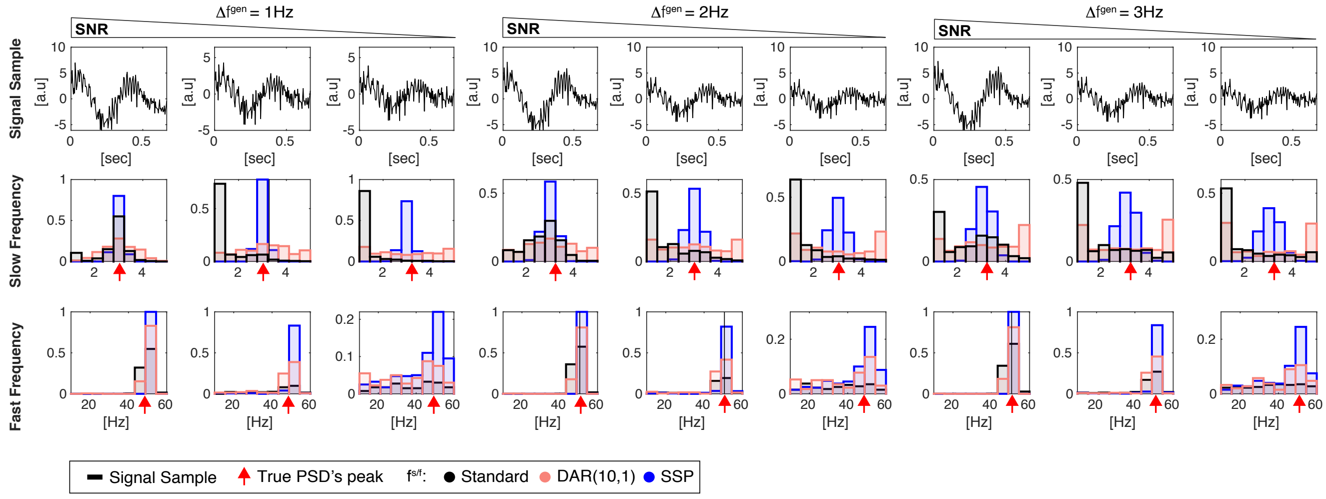

Figure S.8. Slow and Fast oscillation recovery using different algorithm: standard methods (black), DAR (pink) and SSP (blue). 400 windows of 2 seconds were generated with: a slow oscillation (filtered from white noise around $f_s = 3$Hz with bandwidth Δf_s^{gen} and normalized to standard deviation σ_s) and a modulated fast oscillation ($\phi^{\text{mod}} = -\pi/3$, modeled with a sinusoid $f_s = 50$Hz and normalized to σ_f). We added unit normalized Gaussian noise and we used 3 Signal To Noise Ratio (SNR) conditions ($(\sigma_s, \sigma_s) = (2, 1.5)$, $(1, 0.6)$ and $(0.7, 0.3)$). We report typical signal traces for the different conditions (top), slow oscillation recovery alongside the true slow frequency PSD (middle), and fast frequency recovery (bottom). The red arrow indicates the true multitaper PSD (TW=4, K=5 tapers) peak for each oscillation.
